# Examining the role of person-to-person transmission during a verocytotoxigenic *Escherichia coli* outbreak in Ontario, Canada

**DOI:** 10.1186/s13104-022-06075-3

**Published:** 2022-05-21

**Authors:** Roksolana Hovdey, Jan M. Sargeant, David N. Fisman, Amy L. Greer

**Affiliations:** 1 Department of Population Medicine, Ontario Veterinary College, University of Guelph, Guelph, ON Canada; 2 Centre for Public Health and Zoonoses, Ontario Veterinary College, University of Guelph, Guelph, ON Canada; 3 Department of Epidemiology, Dalla Lana School of Public Health, University of Toronto, Toronto, ON Canada

**Keywords:** Disease modelling, Disease transmission, Enteric infections, *Escherichia coli*, Modelling, Multi-chain transmission, Outbreak, Public health, VTEC

## Abstract

**Objective:**

Person-to-person transmission can occur during outbreaks of verotoxigenic *Escherichia coli* (VTEC), however the impact of this transmission route is not well understood. This study aimed to examine the role of person-to-person transmission during a VTEC outbreak, and how targeting this route may reduce outbreak size. A deterministic compartmental model describing a VTEC outbreak was constructed and fit to data from a 2008 outbreak in Ontario, Canada. Using the best-fit model, simulations were run to calculate the: reduction in transmission rate after implementing interventions, proportion of cases infected through both transmission routes, and number of cases prevented by interventions. Latin hypercube sensitivity analysis was conducted to examine the sensitivity of the outbreak size to the model parameters.

**Results:**

Based on the best-fit model, ~ 14.25% of the cases likely arose due to person-to-person transmission. Interventions reduced this transmission rate by ~ 73%, causing a reduction in outbreak size of ~ 17% (47 cases). Sensitivity analysis showed that the model was highly sensitive to changes in all parameters of the model. The model demonstrates that person-to-person could be an important transmission route during VTEC outbreaks. Targeting this route of transmission through hand hygiene and work exclusions could reduce the final outbreak size.

**Supplementary Information:**

The online version contains supplementary material available at 10.1186/s13104-022-06075-3.

## Introduction

Verotoxigenic *Escherichia coli* (VTEC) are pathogens that can cause enteric illness in humans (potentially causing lifelong disability and death), and have been implicated in large outbreaks, including a spinach outbreak in 2006 that affected > 200 people [1–5]. During outbreaks, cases arise from a combination of point source (e.g., ingesting contaminated food/water) and secondary (typically person-to-person) transmission [1, 2, 4, 6–12]. There are knowledge gaps related to the relative contribution of different transmission routes, particularly person-to-person transmission (ppt) in outbreaks [13, 14]. This may be due to challenges associated with ascertaining the source of infection for every case, and the high rate of under-reporting of enteric infections [13, 14]. Food is a major source of transmission of VTEC infections, and ppt is estimated to cause 10–13% of VTEC infections overall, and approximately 20% of cases within an outbreak [11, 15–17]. It is important to better understand the contribution of the different transmission routes to strengthen and target disease prevention and control strategies. Disease modelling has been used to investigate the impact of different transmission routes and interventions on disease dynamics, with one model of a VTEC outbreak estimating that reducing ppt could have reduced the outbreak size by 5–11% [7–9, 18, 19].

The objectives of this study were to: (1) examine the role of ppt in a 2007 VTEC outbreak in Ontario, Canada, and (2) estimate the proportion of cases that were likely prevented through public health interventions targeting ppt. Understanding the impact of person to person transmission in food-borne disease outbreaks can better inform our approach to foodborne disease prevention and control.

## Main text

### Methods

#### Outbreak data

Data for this project were extracted from a report on a foodborne outbreak of VTEC O157 traced to a restaurant in Ontario, Canada in 2008, that infected 235 people [20]. While there was no confirmed source, the suspected food item was shipped to the restaurant approximately one week prior to the initial outbreak detection. Therefore, only cases with a symptom onset date after shipment of the suspected source were included in the dataset (n = 225, 95.7%). Day 1 of the outbreak was considered the first case occurrence after this shipment (Additional file 1 contains the epidemic curve of the outbreak). The public health unit (PHU) was first notified on day 9 of the outbreak. The suspected outbreak source was closed to customers on the evening of day 10. Following closure, communication was sent to the public within the region, disseminating outbreak information and educational materials for preventing ppt. The PHU also directly provided this education to cases, and recommended isolation from workplaces while clinically ill. Cases that worked in food-service settings or daycares were required to isolate from work.

#### Model structure

**Fig. 1:**
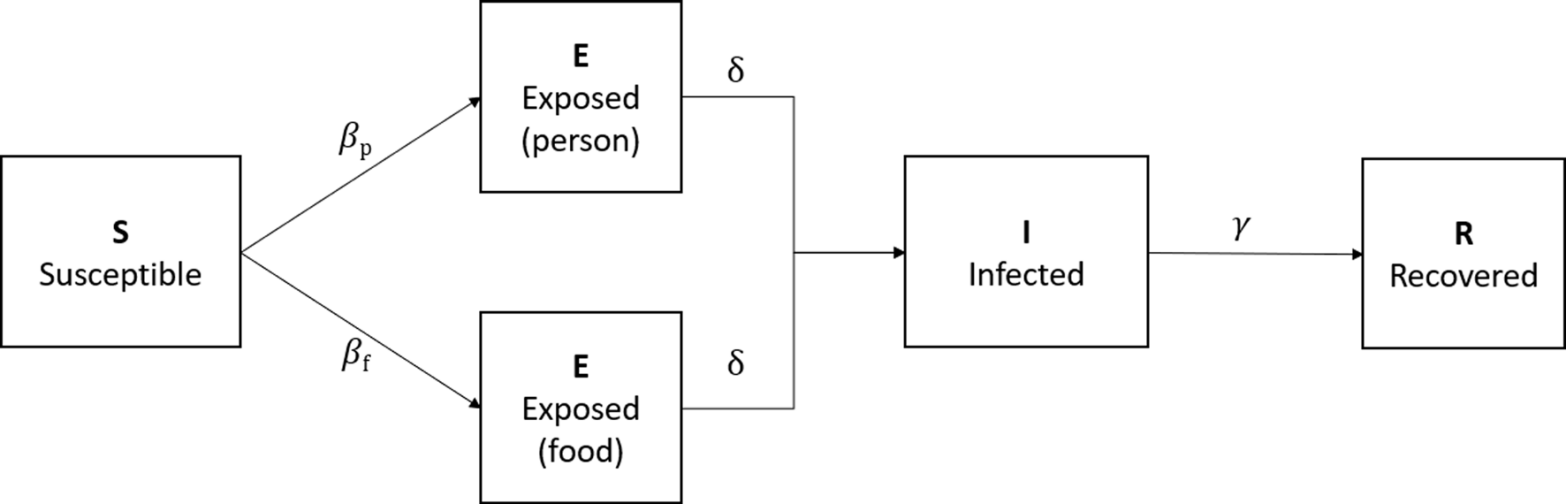
Compartment model for Verocytotoxigenic *Escherichia coli* (VTEC) transmission in humans. Susceptible people (S) are first exposed to VTEC through food (Ef) at a rate of β_f_ or through infected people (Ep) at a rate of β_p_. They become clinically infected (I) and infectious to others at a rate inversely proportional to the incubation period ($\delta$). Infected individuals become recovered (R) from infection at a rate inversely proportional to the duration of infection ($\gamma$)

A deterministic compartment model, informed by the structure of Seto et al. (2007), was developed to describe this outbreak of VTEC (Fig. 1). The model was comprised of five states, Susceptible (S), exposed through food but not infectious (E_f_), exposed through an infected person but not infectious (E_p_), clinically ill and infectious (I), and recovered (R). Susceptible individuals could become exposed to VTEC through contact with contaminated food (β_f_), or through ppt (β_p_). Once exposed, individuals became clinically ill at a rate inversely proportional to the incubation period ($\delta$). Infected individuals remained infectious for the duration of their clinical symptoms, after which they recovered from infection (at a rate of $\gamma$, inversely proportional to the duration of infection) and became immune to reinfection. The disease transmission process is represented by the following differential equations:

$$\frac{dS}{dt}=-\beta_{\text{f}} \text{S}-\beta_{\text{p}}\text{ SI}$$

$$\frac{d{E}_{f}}{dt}=\beta_{\text{f}} \text{S}-\delta \text{E}{\text{f}}$$

$$\frac{d{E}_{p}}{dt}= \beta_{\text{p}}\text{ SI}-\delta \text{E}{\text{p}}$$

$$\frac{dI}{dt}=\delta \left(\text{E}{\text{f}}+\text{E}{\text{p}}\right)-\text{I}\gamma$$

$$\frac{dR}{dt}=\text{I}\gamma$$

Model parameter values can be found in Table 1. It was assumed that all residents of the PHU during the outbreak were susceptible to infection (with homogenous mixing within this population). Due to the short outbreak duration, population demographics (i.e., births/deaths) were not included in the model. Due to the knowledge gaps around asymptomatic VTEC infections, our model assumes that all cases become symptomatic [2, 12, 21, 22].

**Table 1:** Model parameters used (with values obtained from the literature) and best-fit parameters from both scenarios

Symbol	Definition	Value (source)	Range used for LHS sensitivity analysis
Scenario 1			
β_f_	Foodborne transmission rate	4.24 × 10^–4^ (fit to observed data)	(± 25%)
β_p_	Person-to-person transmission rate	2.88 × 10^–7^ (fit to observed data)	(± 50%)
*δ*	Incubation period^a^	3.5 days [2, 3, 7, 12, 25, 28]	(2–10) days
*γ*	Duration of infection^a^	6 days [7, 25, 28]	(5–10) days
Scenario 2			
β_f_	Foodborne transmission rate	4.24 × 10^–4^ (fit to observed data)	(± 25%)
β_p1_	Pre-intervention person-to-person transmission rate	1.10 × 10^–6^ (fit to observed data)	(± 50%)
β_p2_	Post-intervention person-to-person transmission rate	2.88 × 10^–7^ (fit to observed data)	(± 50%)
*δ*	Incubation period^a^	3.5 days [2, 3, 7, 12, 25, 28]	(2–10) days
*γ*	Duration of infection^a^	6 days [7, 25, 28]	(5–10) days

^a^Inverse value is used as a rate in the model

There were multiple interventions implemented during the outbreak response, targeting both the primary source and secondary transmission. The restaurant closure was modelled by assuming that transmission of VTEC through food (βf) decreased to zero after day 10. The public health interventions targeted at reducing ppt (e.g., work exclusion, public health messaging) were modelled as one intervention that reduced ppt after day 10.

#### Model fitting and outcomes

The model was fit to outbreak incident cases by considering two different scenarios: (1) where ppt rate remained constant for the duration of the outbreak, and (2) where the transmission rates differed prior-to and after the implementation of the intervention (details can be found in Additional file 2). The parameter values estimated for scenario 1 were used in scenario 2 (with the ppt rate from scenario 1 used as the post-intervention transmission rate in scenario 2) to estimate the ppt rate prior to public health interventions. The model was calibrated using the mle2 function in R from the bbmle package to estimate parameters using maximum likelihood estimation [23].

Using the best-fit model scenario, we investigated several outcomes: We calculated the proportion of model-simulated cases that likely arose due to ppt. We compared the ppt rate pre- and post-intervention in scenario 2 to estimate how much the intervention reduced the ppt rate. To calculate the number of cases averted by targeting ppt, we compared the outbreak size from the best-fit model scenario to a simulated scenario where no public health interventions targeting ppt occurred after the closure of the point source.

#### Sensitivity analysis

A Latin Hypercube sensitivity analysis was performed for all model parameters. Ranges for the analysis can be found in Table 1. Partial rank correlation coefficients were calculated to investigate how changes to the parameters influenced the model projected outbreak size.

### Results

#### Model fit

**Fig. 2:**
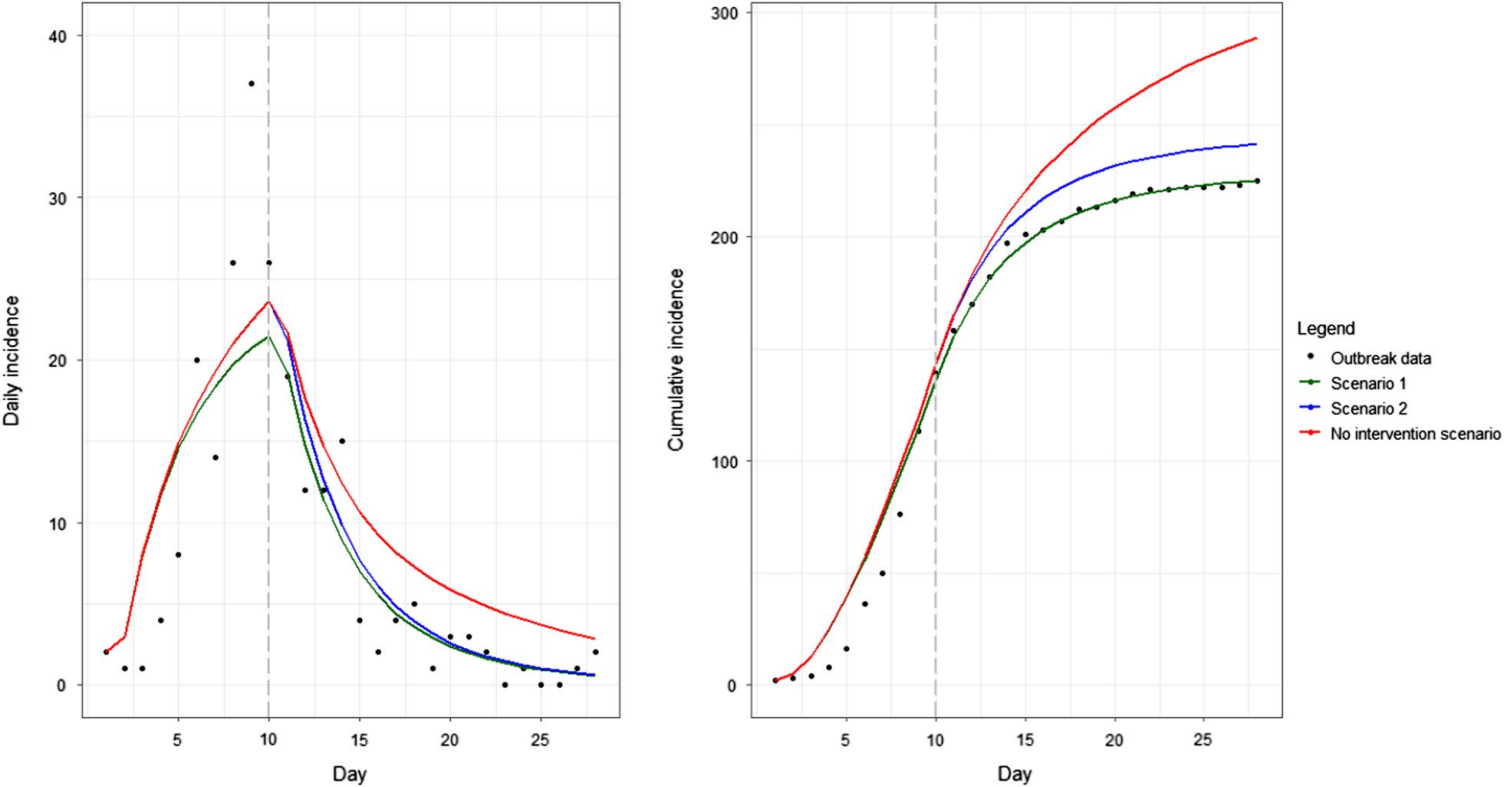
Simulation of all model scenarios compared to the observed outbreak data. Grey line indicates the start date of all interventions (point source closure, and initiation of public health interventions targeted at reducing person-to-person transmission)

Table 1 contains the best fit parameters from the proposed model. Based on maximum likelihood estimation, scenario 2 was a better fit to the observed data. However, the model was not able to capture the peak incidence that occurred on day 9 and appeared to fit poorly prior to the intervention start date (Fig. 2). While the scenario 1 simulation estimated the same outbreak size as the observed data, the peak incidence occurred one day later and underestimated the observed peak (specific details can be found in Additional file 3). Similarly, scenario 2 simulated the peak incidence one day later and underestimated the observed outbreak peak. However, this simulation estimated a larger outbreak size. For both scenarios, a greater proportion of outbreak cases were attributed to ppt (scenario 1: 8.00%, scenario 2: 14.25%) compared to the observed data (4.88%).

#### Model intervention

Based on the best-fit model parameters in scenario 2, the ppt rate post-intervention was 73.83% lower than the rate pre-intervention. In the “no intervention” scenario, an outbreak size of 289 cases was simulated. Therefore, the decrease in the ppt rate in the best-fit scenario translated to a reduction in outbreak size by 16.47%.

#### Sensitivity analysis

Additional file 4 contains the results of the Latin hypercube sensitivity analysis as partial rank correlation coefficients (PRCCs). The model was quite sensitive to changes in all model parameters (PRCC: > 0.5 or < − 0.5). Increasing the values of the pre- and post- intervention ppt rates, the foodborne transmission rate, and the incubation rate parameter translated to an increase in the VTEC outbreak size, while increasing the rate of recovery led to a decrease in the final VTEC outbreak size.

### Discussion

We used a mathematical model to estimate the relative contribution of ppt during a VTEC outbreak and quantified the impact public health interventions had on the outbreak size. The results of our study highlight a multi-route transmission chain during this outbreak. We determined that ppt may play a slightly larger role in VTEC outbreaks than previously suggested, and that interventions targeting this route appear to reduce outbreak size [11, 15–17].

Despite scenario 2 having a better fit to the observed data, scenario 1 was also similar. Given our understanding of the interventions implemented, it is not realistic to assume that ppt remained constant throughout the outbreak. The overall lack of fit may be due to some cases in the observed outbreak being misclassified as part of the outbreak. It is also likely that the pre-intervention ppt was underestimated due to our model fitting process. As the constant ppt rate is essentially an ‘average’ of the pre- and post-intervention transmission rates, setting that value as the post-intervention transmission rate likely led to an underestimation of the pre-intervention transmission rate.

The proportion of secondary cases estimated in our model was higher than reported in the outbreak data, suggesting that some cases may have been misclassified as primary cases [20]. The proportion of secondary cases estimated by our best-fit model is in line with current estimates derived through expert elicitations and statistical analysis [11, 15–17, 24]. These estimates reported ppt contributing 10–13% of VTEC infections overall, and around 20% of infections within an outbreak [11, 15–17, 24]. Other mathematical models also found similar proportions of ppt during enteric outbreaks [7, 9]. A similar VTEC model was only able to fit well under the assumption that ppt contributed between 12 and 25% of the outbreak cases in their study [7]. Additionally, outbreaks with a younger median age group were associated with a high proportion of secondary cases [17]. It is possible that the lower estimate in our study could be due to a higher median age; however more information on the demographics of cases was not available.

Our analysis suggests that interventions targeting ppt reduced outbreak size by approximately 16%. This reduction is higher than a similar study, which reported reductions to outbreak size by 7–11%; however, that study examined a theoretical intervention, while we examined a real intervention [7]. Lastly, differences between results may be due to differences in model structure. There is further evidence from studies of other infectious diseases that interventions focused on reducing ppt (e.g., isolation, hand-hygiene) can reduce transmission risk and final outbreak size [7, 25–34]. One study estimated that reducing ppt resulted in a 23% reduction in the cumulative influenza attack rate [27]. Mathematical models have also shown that media campaigns can lead to behavioural change and cause a reduction in secondary transmission [28–32].

Based on the sensitivity analysis, the model was highly sensitive to changes in all of the parameters in the model, indicating that changing parameter values would significantly change outbreak size. Therefore, all parameter values must fall within a narrow range to fit to the observed data.

## Conclusion

We found that mathematical modelling could be used to estimate the relative contribution of different transmission parameters in a disease outbreak. Additionally, we found that targeting the ppt route during enteric outbreaks could be an effective method for reducing the outbreak size. Future VTEC outbreak management should include strategies to prevent secondary transmission. Prevention methods can include isolation of infected persons and improved hygiene techniques. Future modelling studies could investigate the impact of asymptomatic infections during outbreaks, estimate transmission of VTEC within-households, and examine the person-food transmission route.

## Limitations

- The model did not consider asymptomatic transmission, age-structure, or person-food transmission after the initial exposure occurred.
- It was assumed that the population of the PHU was equally at risk of infection. Due to the source of the outbreak, and since VTEC is commonly transmitted within households, it is likely that the true population at risk was smaller [2, 10, 12, 17, 35].
- It was assumed that both transmission rates were constant. It is likely that the risk of exposure changed over time; however, details on exact exposures were unknown. Similarly, since interventions targeting ppt rely on human behaviour, the effects of these interventions likely also vary in time [26, 28–31, 34]. The model could be missing this additional variability.
- All public health interventions targeting ppt were modelled as one all-encompassing intervention. Therefore, we were not able to examine the impact that each individual intervention had on reducing ppt.
- The process of estimating the ppt transmission rate may have led to an underestimation of the pre-intervention ppt rate, and an overestimation in the post-intervention transmission rate.

## Supplementary Information

**Additional file 1. **Plot of the Verocytotoxigenic *Escherichia coli* (VTEC) outbreak in Ontario, Canada. Arrows indicate relevant dates during the outbreak.

**Additional file 2. **Table describing the two different scenarios that represent possible explanations for the observed outbreak data.

**Additional file 3. **Characteristic of the observed outbreak data, compared to the two simulated model scenarios and the “no intervention” simulation.

**Additional file 4. **Results of the Latin hypercube sensitivity analysis for all parameters in the model, represented as partial rank correlation coefficients (PRCCs). The PRCCs represent the effect of varying each parameter on the final outbreak size.

## Data Availability

The dataset analysed during the current study was derived from the investigative report published by the local health unit [20]. The report is available at: https://www.deslibris.ca/ID/219086.

## Abbreviations

VTEC: Verocytotoxigenic *Escherichia coli*
ppt: Person-to-person transmission
PHU: Public health unit
PRCC: Partial rank correlation coefficient
