# Supplementary figures and images for: Examining the role of person-to-person transmission during a verocytotoxigenic Escherichia coli outbreak in Ontario, Canada
Source: BMC Res Notes. 2022 May 21;15:187. doi: 10.1186/s13104-022-06075-3 (PMC9123793; doi:10.1186/s13104-022-06075-3)

**
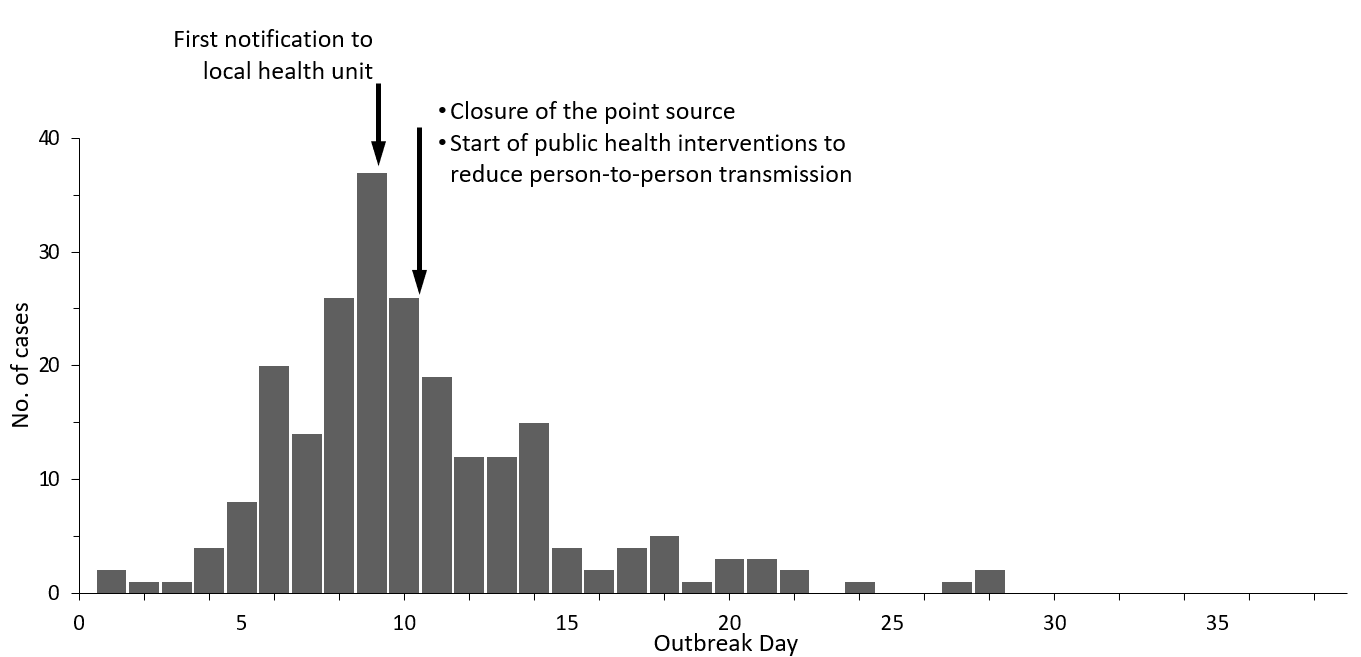
**

Supplement: Supplementary file 1 — Additional file 1. Plot of the Verocytotoxigenic Escherichia coli (VTEC) outbreak in Ontario, Canada. Arrows indicate relevant dates during the outbreak. [file 13104_2022_6075_MOESM1_ESM.docx]

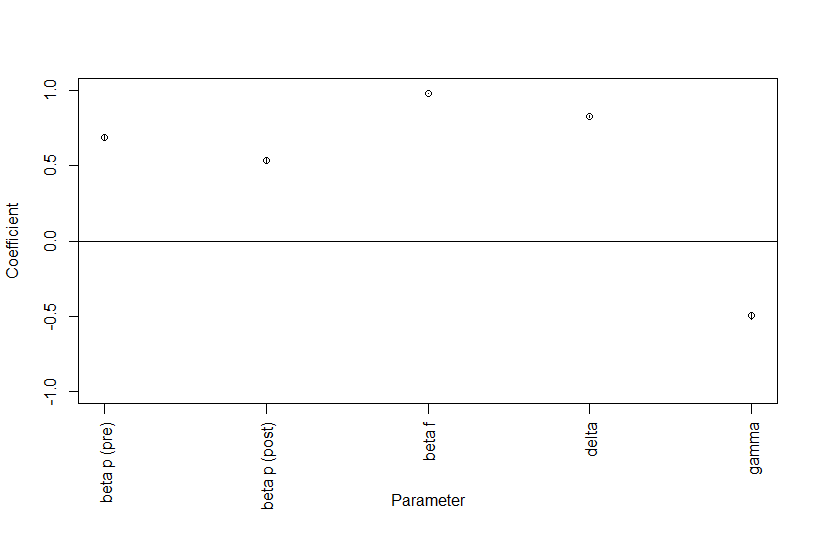

Supplement: Supplementary file 4 — Additional file 4. Results of the Latin hypercube sensitivity analysis for all parameters in the model, represented as partial rank correlation coefficients (PRCCs). The PRCCs represent the effect of varying each parameter on the final outbreak size. [file 13104_2022_6075_MOESM4_ESM.docx]
