# Supplementary material for: Examining the role of person-to-person transmission during a verocytotoxigenic Escherichia coli outbreak in Ontario, Canada
Source: BMC Res Notes. 2022 May 21;15:187. doi: 10.1186/s13104-022-06075-3 (PMC9123793; doi:10.1186/s13104-022-06075-3)
Supplement: Supplementary file 2 — Additional file 2. Table describing the two different scenarios that represent possible explanations for the observed outbreak data. [file 13104_2022_6075_MOESM2_ESM.docx]

| **Scenario** | **Description of Scenario** |
| --- | --- |
| 1 | - Foodborne transmission (β_f_) was present until restaurant closure (day 10) after which β_f_ = 0 - Person‑to‑person transmission (β_p_) was present at a constant rate over the entire duration of the outbreak |
| 2 | - Foodborne transmission (β_f_) was present until restaurant closure (day 10) after which β_f_ = 0 - Person‑to‑person transmission (β_p_) was present throughout the entire outbreak, but the rate was reduced after day 10 as a result of the public health interventions that were deployed. |
