# Supplementary material for: Examining the role of person-to-person transmission during a verocytotoxigenic Escherichia coli outbreak in Ontario, Canada
Source: BMC Res Notes. 2022 May 21;15:187. doi: 10.1186/s13104-022-06075-3 (PMC9123793; doi:10.1186/s13104-022-06075-3)
Supplement: Supplementary file 3 — Additional file 3. Characteristic of the observed outbreak data, compared to the two simulated model scenarios and the “no intervention” simulation. [file 13104_2022_6075_MOESM3_ESM.docx]

| **Type of data** | **Final outbreak size (difference)** | **Peak Daily Incidence (difference)** | **Day of peak incidence (difference)** | **Number of cases infected due to person‑to‑person transmission (%)** |
| --- | --- | --- | --- | --- |
| Outbreak data | 225 | 37 | 8 | 11 (4.89) |
| Scenario 1 - simulation | 225 (0) | 22 (-15) | 9 (+1) | 18 (8.00) |
| Scenario 2 - simulation | 242 (+17) | 24 (-13) | 9 (+1) | 34 (14.25) |
